# The Dynamics of Embolism Refilling in Abscisic Acid (ABA)-Deficient Tomato Plants

**DOI:** 10.3390/ijms14010359

**Published:** 2012-12-24

**Authors:** Francesca Secchi, Irene Perrone, Walter Chitarra, Anna K. Zwieniecka, Claudio Lovisolo, Maciej A. Zwieniecki

**Affiliations:** 1 UC Davis, PES, One Shields Avenue, Davis, CA 95616, USA; E-Mail: mzwienie@ucdavis.edu; 2 Arnold Arboretum of Harvard University, 1300 Centre St, Boston, MA 02131, USA; E-Mail: irene.perrone@plantphys.umu.se; 3 Department of Agricultural, Forest and Food Sciences (AGRIFORFOOD), University of Turin, Via Leonardo da Vinci, 44, Grugliasco 10095, Italy; E-Mails: walter.chitarra@unito.it (W.C.); claudio.lovisolo@unito.it (C.L.); 4 BioLabs, Harvard University, 16 Divinity Ave, Cambridge, MA 02138, USA; E-Mail: azwienie@oeb.harvard.edu

**Keywords:** abscisic acid, ABA-deficient tomato, starch, vessel embolism, water stress

## Abstract

Plants are in danger of embolism formation in xylem vessels when the balance between water transport capacity and transpirational demand is compromised. To maintain this delicate balance, plants must regulate the rate of transpiration and, if necessary, restore water transport in embolized vessels. Abscisic acid (ABA) is the dominant long-distance signal responsible for plant response to stress, and it is possible that it plays a role in the embolism/refilling cycle. To test this idea, a temporal analysis of embolism and refilling dynamics, transpiration rate and starch content was performed on ABA-deficient mutant tomato plants. ABA-deficient mutants were more vulnerable to embolism formation than wild-type plants, and application of exogenous ABA had no effect on vulnerability. However, mutant plants treated with exogenous ABA had lower stomatal conductance and reduced starch content in the xylem parenchyma cells. The lower starch content could have an indirect effect on the plant’s refilling activity. The results confirm that plants with high starch content (moderately stressed mutant plants) were more likely to recover from loss of water transport capacity than plants with low starch content (mutant plants with application of exogenous ABA) or plants experiencing severe water stress. This study demonstrates that ABA most likely does not play any direct role in embolism refilling, but through the modulation of carbohydrate content, it could influence the plant’s capacity for refilling.

## 1. Introduction

Plants subject to water stress are susceptible to cavitation and the formation of embolism in xylem conduits [1]. The presence of embolism results in the loss of xylem capacity to transport water to meet the transpirational demand [2,3] and could lead to desiccation or death of photosynthetic tissue in the event of runaway cavitation [4]. Such imbalance between transport capacity and transpirational demand may occur due to the dynamic changes in the plant micro-environment, and thus, plants have evolved multiple structural properties and physiological processes to maintain the balance between transpirational demand and water transport capacity. In general, the mechanisms used to maintain this balance are limited to either control of transpiration rate or development/maintenance of xylem transport capacity; both processes serving to reduce tension in the xylem [2,3,5,6].

Long-term responses of a plant challenged with increased evaporative losses would mostly be developmental and might include an increase in the ratio of xylem area to leaf area through growth of xylem tissue or reduction in canopy size and an increase of root:shoot ratio. Rapid changes in water demand due to changing humidity, light exposure and temperature require mechanisms that respond in much shorter time scales. These short-term responses rely on stomatal movement [7] and on the plant’s ability to restore water transport in embolized conduits [8–11]. The maintenance of the short-term balance between supply and loss of water is mostly under the control of stomata, which respond to multiple biological and physical factors, like light, CO_2_, vapor pressure, plant water potential and ABA [12–16]. The role of embolism removal and restoration of the xylem transport capacity is much less understood, although the process might be partially affected by the same stress related factors, thus enforcing a coordinated plant behavior [17].

Abscisic acid (ABA) has been considered the dominant long-distance chemical signal mediating the effects of soil drought on stomatal conductance [18–20]. ABA is synthesized both in the leaves and the roots. It can be transported rapidly through the plant via the xylem and the phloem [21,22]. Its production is up-regulated during water stress [23], and it was shown to be responsible for multiple stress-related plant reactions, such as osmotic adjustment, ion transport and stomatal closure [24,25]. Previously, the role of ABA in stomatal movement was tested on mutant lines of tomato plants characterized by reduced levels of ABA production in response to soil drying, revealing that stomata were unresponsive to moderate water stress [26]; however, external application of ABA was shown to restore the stomatal function [27].

The details of the role of ABA in stomatal closure are still being studied, but we can expect that the accumulation of the apoplastic ABA in evaporation sites of leaves can trigger specific signaling pathways that result in turgor loss of guard cells and reduction in stomatal conductance. The loss of turgor would require that the guard cells change their osmotic potential in order to trigger the efflux of water from cells. The mechanism of stomatal closure in response to ABA might share similarities with an osmotic model of embolism refilling [17]. The osmotic refilling model suggests that embolism refilling occurs, due to active pumping of osmolytes (e.g., sugars derived from depolymerization of starch stored in xylem parenchyma) into water droplets in the embolized xylem, and subsequent passive influx of water through aquaporins along the water potential gradient. The action of ABA might share some similarities with this model; specifically, the presence of ABA generates efflux of water from guard cells, possibly due to stimulation of osmotic release (e.g., alteration of carbon partitioning between starch and soluble sugars), and thus, if parenchyma cells respond to ABA in the same way, there is a potential for ABA to stimulate refilling by triggering release of water from cells in the vicinity of vessels.

Water transport activity is moderated by the activity of aquaporins [28,29], the function of which is probably interconnected with abscisic acid signal transduction [30]. Thus, ABA may be able to increase trans-cellular water flow by upregulating activity of aquaporins [29,31–33]. Recently, an increase of ABA concentration was observed in leaves of grapevine plants recovering from drought stress under high transpiration rates that coincided with high rates of recovery from water stress, including restoration of water potential, embolism refilling and stomatal conductance [34]. Interaction between ABA and hydraulic was also described by Pantin *et al*., showing that ABA remotely controls stomatal response by changing hydraulic conductance in the leaf, upstream of stomata [35].

That ABA may be involved in the embolism/refilling cycle is attractive, as ABA signaling (increasing concentration in response to water stress) and physiological response would be identical in stem and leaves. A single stimulus for both stomatal closure and initiation of embolism refilling would simultaneously help balance plant transport capacity and transpiration rate—two aspects of plant water status not previously linked by a chemical signal. Here, we present a study that aims to experimentally test the possible role of ABA in xylem embolism/refilling cycles.

## 2. Results

**Figure 1:**
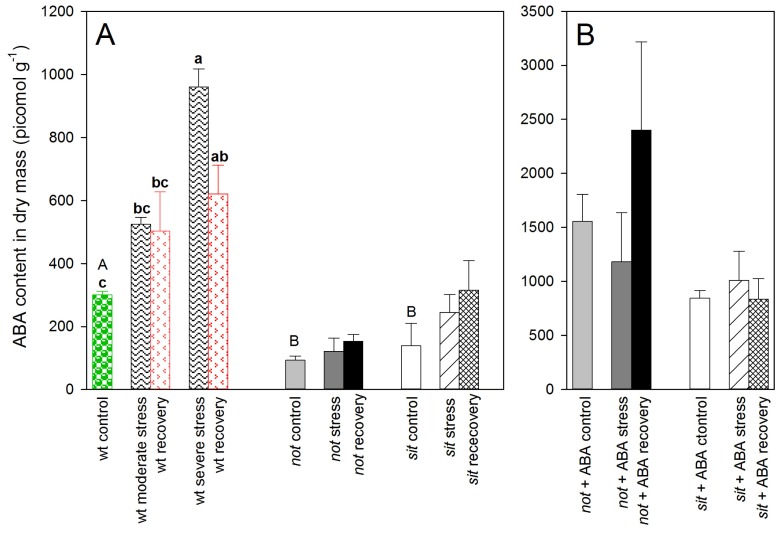
(**A**) Concentration of Abscisic Acid (ABA) in petioles of *wt* and two ABA-deficient mutant lines (*not* and *sit*) at different stages of water stress and recovery. Capital letters above bars of control plants denote homogeneous groups with *p* < 0.05 using the Duncan test (error bars denote SE). Lower case letters denote homogeneous groups between treatments within the *wt* plants. (**B**) Concentration of ABA in petioles of *not* and *sit* plants after repeated application of exogenous ABA.

We compared tomato wild-types with two different ABA-deficient mutant tomato lines: the *not* mutants, having a defect in a key control step in ABA biosynthesis, and the *sit* mutants, impaired in ABA-aldehyde oxidation and accumulating trans-ABA-alcohol instead of ABA. The endogenous ABA concentration levels were similar in irrigated *not* and *sit* mutant lines and significantly lower than well-watered wild-type (*wt)* plants. In *wt* tomatoes, this initial difference was later magnified during increasing water stress, while in both mutant lines, the stress treatment had no effect on ABA concentration (Figure 1A). During recovery from stress, the level of ABA dropped in *wt* plants, while ABA concentration did not significantly change in re-watered mutant plants (Figure 1A). The application of exogenous ABA to the leaf surface of ABA-deficient mutants significantly increased the hormone concentration in petioles of treated plants, but again, there was no significant change of ABA concentration in response to the experimental treatments (drought stress or recovery; Figure 1B).

**Figure 2:**
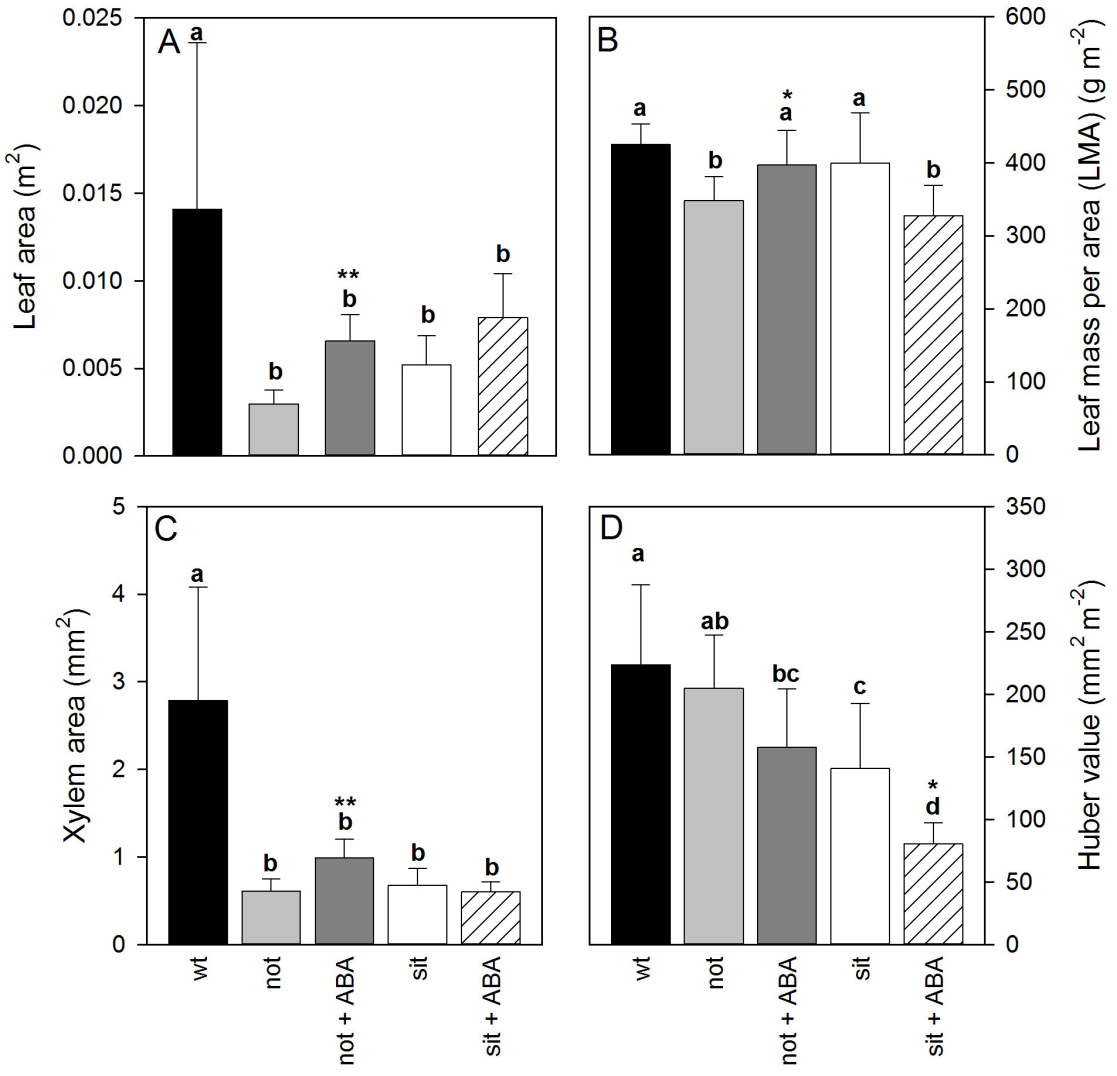
Leaf allometric properties of well-watered plants: (**A**) leaf area, (**B**) leaf mass per area (LMA), (**C**) xylem area at the base of the petiole and (**D**) Huber value expressed as xylem area (mm^2^) divided by the leaf area (m^2^). Letters above the bars denote homogeneous groups with *p* < 0.05 determined using Fisher LSD test (df = 31). Stars above the bars denote presence of a significant difference within each mutant experiment, where * indicates 0.01 < *p* < 0.05 and **p < 0.001, determined using *t*-test.

There was a significant difference in leaf allometry between leaves of *wt* and mutant lines. The leaf and xylem area of *wt* was significantly larger than ABA-deficient plants. The leaf area, leaf mass per area (LMA) and the xylem area of exogenous ABA treated *not* mutants were significantly larger than ABA-deficient *not* plants. Those leaf allometric properties did not change between *sit* mutants (Figure 2A–C). Interestingly, addition of exogenous ABA resulted in developmental changes such that xylem area to leaf area ratio (Huber value) dropped in both mutant lines (Figure 2D); however, only *sit* lines showed a significant decrease (*t*-test *p* < 0.05 df = 12). The Huber value can reflect the load of the transpiration on transport system, and these drops could reflect a reduced g_s_ due to application of ABA. Thus, a decreased load stress on the transport system may allow plants to develop a higher leaf area for a given xylem area. (Statistics for observed significant differences: *not* and *not* + ABA leaf area: df = 14, *t*-value = 6.139 and *p* = 0.000026; *not* and *not* + ABA LMA: df = 14, *t*-value = 2.20807 and *p* = 0.04423; *not* and *not* + ABA xylem area: df = 14, *t*-value = 4.8742 and *p* = 0.00024; *sit* and *sit* + ABA Huber value: df = 10, *t*-value = 2.7223 and *p* = 0.02417; error bars denote SE).

**Figure 3:**
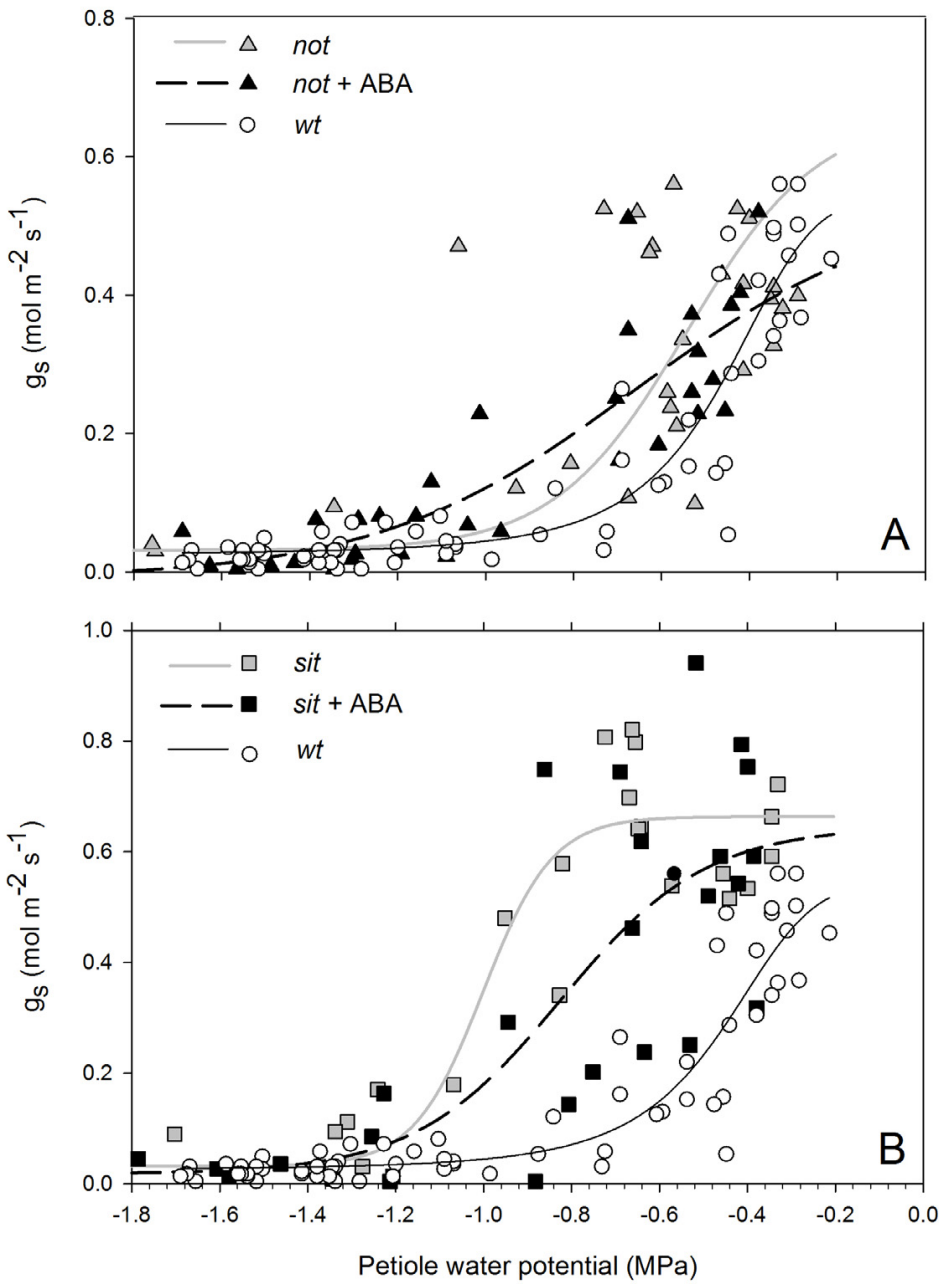
Stomatal conductance (g_s_) of *not* (**A**) and *sit* (**B**) ABA-deficient mutant lines and mutant plants with application of exogenous ABA undergoing more negative petiole water potentials due to increasing water stress. There was no statistical difference between both mutant lines (*not* EC_50_ = −0.703 and *sit* EC_50_ = −0.967 df = 51 *p* = 0.22). However, during stress conditions, *sit* and *wt* plants showed a significantly different stomata response (*sit* EC_50_ = −0.967 and *wt* EC_50_ = −0.455 df = 82 *p* < 0.0001), but no difference was measured between *not* and *wt p*lants (*not* EC_50_ = −0.703 and *wt* EC_50_ = −0.455 df = 92 *p* = 0.22). Solid grey lines (**A**,**B**) show trend lines from ABA-deficient mutants to plants with application of exogenous ABA (dotted black lines)

Stomatal conductance of non-stressed *wt* and *not* mutant plants was ~0.45 (mol m^−2^ s^−1^) and lower than *sit* mutant plants (~0.7 mol m^−2^ s^−1^). Exposure to water stress resulted in a drop of g_s_ in all lines (Figure 3A,B). Wild-type plants had a complete stomatal closure at petiole water potentials of approximately −0.8 MPa. Stomatal conductance of *not* mutants showed a gradual and highly variable trend; while some leaves had stomata fully open at −0.7 MPa, some had stomata fully closed (Figure 3A). Stomatal conductance of the *sit* remained constant around its maximum value until plants reached petiole water potentials of approximately −1.1 MPa, at which point stomata closed with little further reduction in water potential (Figure 3B). In all instances, mutant and *wt* plants had closed stomata at stress levels below −1.2 MPa. Application of exogenous ABA to mutant lines resulted in a moderate shift of stomatal response to petiole water potential. The shift was not significant due to a high variability of the response between leaves (*not* EC_50_ = −0.703 and *not* + ABA EC_50_ = −0.75 df = 61 *p* = 0.39; *sit* EC_50_ = −0.967 and *sit* + ABA EC_50_ = −0.846 df = 47 *p* = 0.27).

**Figure 4:**
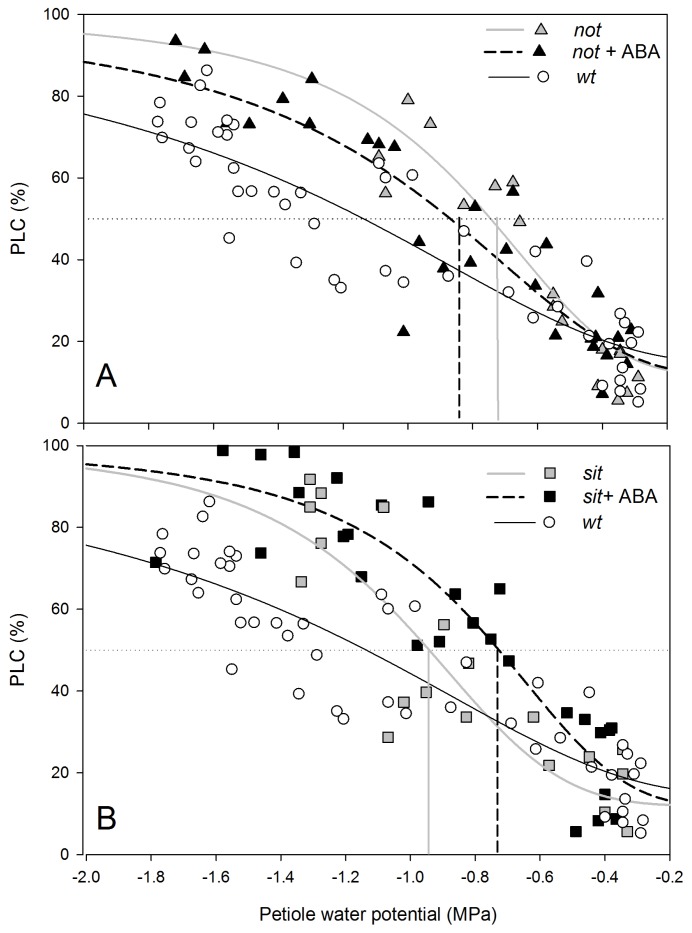
Percent loss of hydraulic conductance (PLC) in petioles of *not* mutants (**A**) and *sit* mutants (**B**), with more negative petiole water potentials due to increasing water stress. There was no statistical difference between both mutant lines (*not* EC_50_ = −0.811 and *sit* EC_50_ = −1.01 df = 37 *p* = 0.15). There was also no significant change in PLC response to application of ABA on mutant plants (*not* EC_50_ = −0.811 and *not* + ABA EC_50_ = −0.965 df = 45 *p* = 0.33; *sit* EC_50_ = −1.01 and *sit* + ABA EC_50_ = −0.793 df = 45 *p* = 0.16), neither between mutant and *wt* plants (*not* EC_50_ = −0.812 and *wt* EC_50_ = −1.33 df = 64 *p* = 0.34; *sit* EC_50_ = −1.01 and *wt* EC_50_ = −1.33 df = 65 *p* = 0.37). Solid grey lines (**A**,**B**) show trend lines from ABA-deficient mutants to plants with application of exogenous ABA (dotted black lines).

Analysis of changes in xylem transport capacity proved that both mutant plants had a higher loss of conductivity in the xylem of petioles than *wt* plants (Figure 4A,B). Embolism vulnerability curves obtained from measurements relating petiole water potential of transpiring plants with PLC showed that a 50% loss of conductivity occurred at around −0.75 MPa for *not* plants and −0.95 MPa for *sit* mutant lines (Figure 4A,B); *i.e.*, before occurrence of full stomatal closure. Almost complete loss of xylem conductance (~80% or more) was observed at −1.2 MPa for both mutant lines. Exogenous application of ABA did not significantly change the vulnerability to embolism in mutant plants (Figure 4A,B), which would be consistent with a lack of changes in sensitivity of stomatal conductance to water stress (Figure 3A,B).

**Figure 5:**
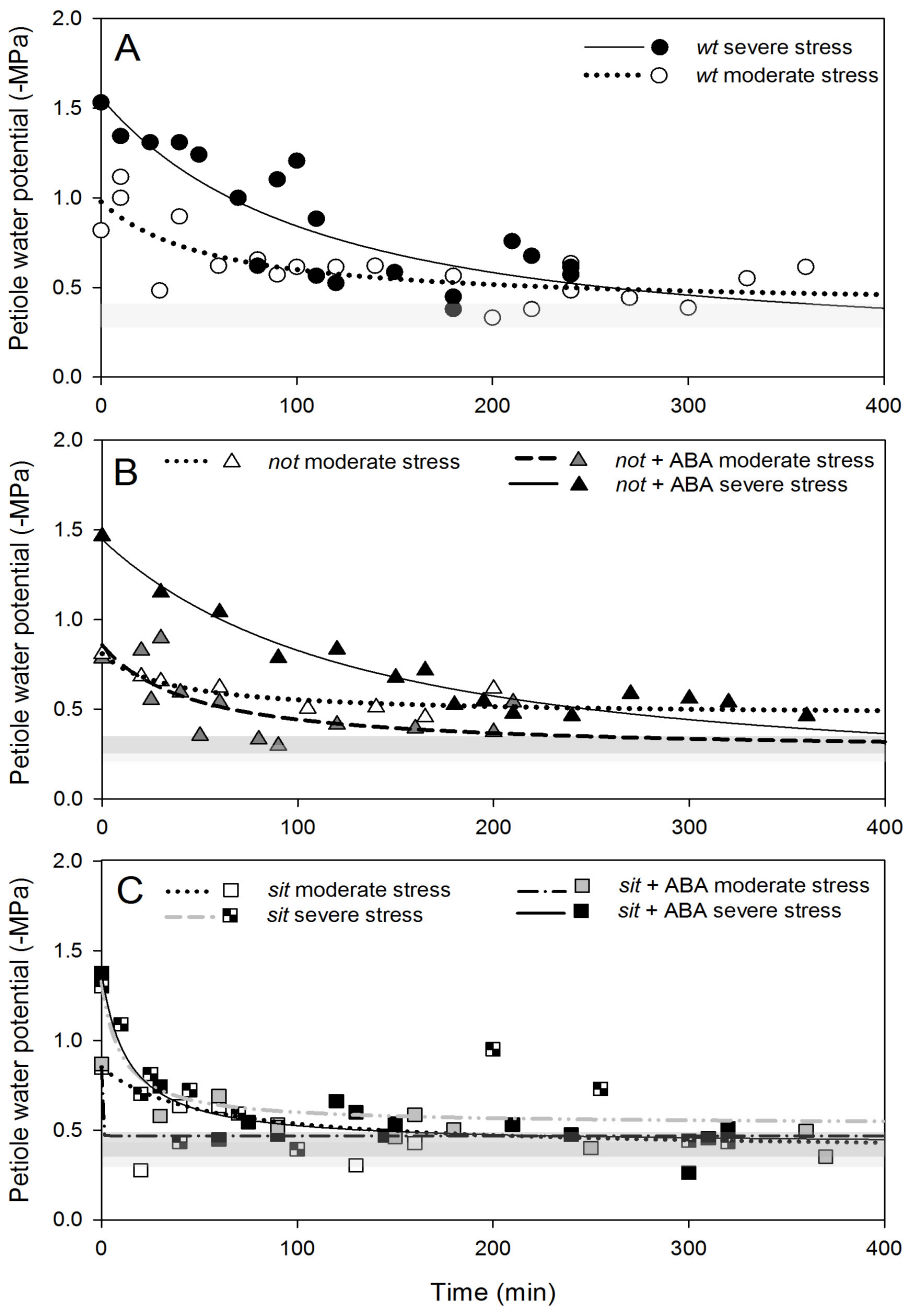
Temporal dynamics of the recovery of petiole water potential following re-watering after exposure to moderate or severe water stress (**A**) *wt*, (**B**) *not* mutants and (**C**) *sit* mutants. Stressed plants were re-watered at time zero, corresponding approximately to 9 am. Fitted lines (exponential decay functions) are provided to focus attention on the general trend of recovery. The shaded grey area width represents average (±SD) petiole water potential, respectively, for well-watered *wt* and ABA-deficient mutants (light grey area) and for well-watered exogenous ABA treated plants (dark grey area).

The basic dynamics of the recovery from water stress involve, amongst others, increasing water potential, stomatal opening and recovery from embolism loss. We studied recovery from two different levels of water stress: moderate (petiole water potentials >(−1.0) MPa) and severe (<(−1.0) MPa). Recovery of petiole water potential occurred in all *wt* and mutant plants in a very similar pattern. Moderately stressed plants returned almost to non-stress levels within 120 min, while severely stressed plants recovered within 400 min (Figure 5A–C). Application of exogenous ABA to mutant plants did not alter the recovery of petiole water potential (Figure 5A–C).

**Figure 6:**
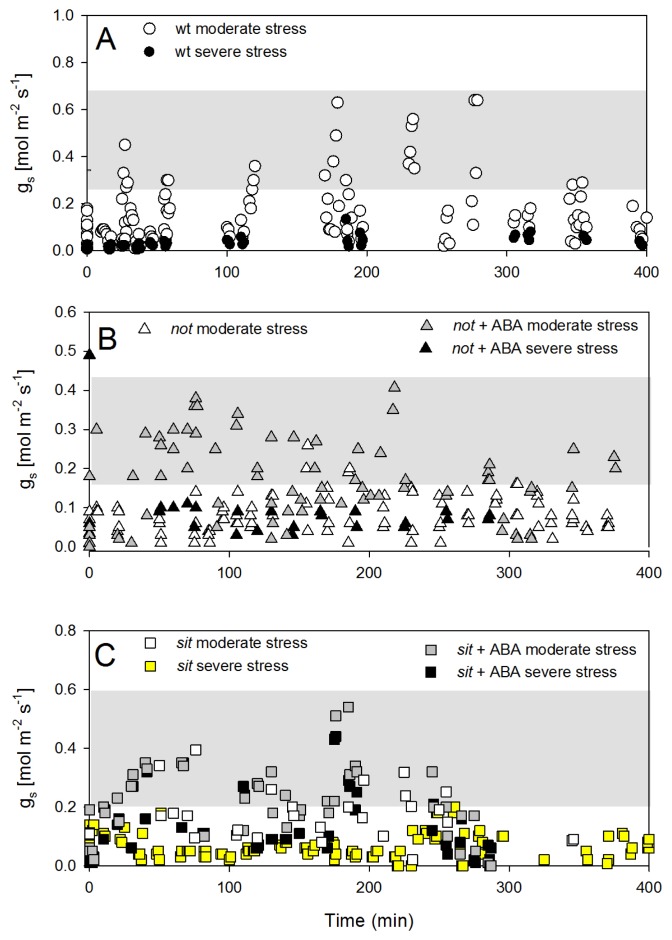
Temporal dynamics of stomatal conductance of well-watered (control) and plants recovering from water stress for (**A**) *wt*, (**B**) *not,* and (**C**) *sit* mutant lines. Stressed plants were re-watered at time zero, corresponding approximately to 9 am. Shaded grey area width represents average (±SD) g_s_ for well-watered *wt* and ABA-deficient mutants (control plants). Based on data presented in Figure 3, there was no difference between non-treated and exogenous ABA treated well-watered mutants. Thus, for this experiment, well-watered mutants non-treated with ABA were used as controls.

The recovery of stomatal conductance was not as pronounced as petiole water potential. Wild-type plants showed only partial recovery following the re-watering and maintained low stomatal conductance, despite the return of petiole water potential close to those of well-watered plants (Figure 6A). Interestingly, there was no recovery of stomatal conductance in both *not* and *sit* mutant plants, but when mutants were treated with exogenous ABA, there was a partial (although statistically significant) recovery of g_s_ (Figure 6B,C). This observation may suggest that exogenous ABA application restores some sensitivity of mutant lines to changes in leaf water potential.

**Figure 7:**
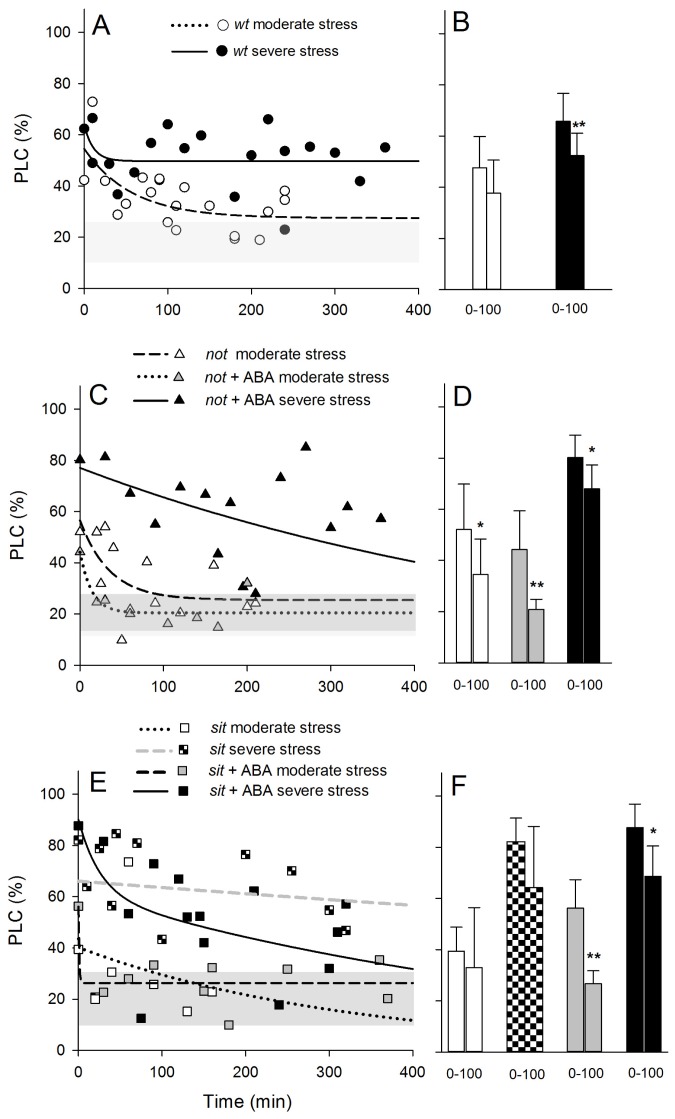
Dynamics of the recovery of PLC following re-watering from moderate and severe stress levels for *wt* (**A**,**B**), *not* (**C**,**D**) and *sit* mutants (**E**,**F**). Fitted lines (exponential decay functions) are provided to focus attention on the general trend of recovery. Shaded grey area width represents average (±SD) PLC, respectively, for well-watered *wt* and ABA-deficient mutants (light grey area) and well-watered exogenous ABA-treated mutants (dark grey area). Stars above the bars denote presence of significant difference in each group within 0 and 100 min after re-watering, where * indicates 0.01 < *p* < 0.05 and **p < 0.001, determined using *t*-test (**B**,**D**,**F**). (Statistics for observed significant differences: *wt* severe stress: df = 25, *t*-value = 4.23 and *p* = 0.00027; *not* moderate stress: df = 17, *t*-value = 2.167 and *p* = 0.04; *not* + ABA moderate stress: df = 15, *t*-value = 3.337 and *p* = 0.004; *not +* ABA severe stress: df = 12, *t*-value = 2.473 and *p* = 0.028; *sit* + ABA moderate stress: df = 12, *t*-value = 5.035 and *p* = 0.00029; *sit* + ABA severe stress: df = 12, *t*-value = 3.265 and *p* = 0.0067).

Patterns of PLC recovery were similar to those observed in the water potential recovery following re-watering. Within 200 min, all moderately stressed plants showed a relatively strong pattern of refilling of embolized vessels (Figure 7A–C), and the measured PLC of *not* mutant plants was already significantly different within 100 min after re-watering (Figure 7D). Such significant recovery was not observed in *sit* and *wt* plants (Figure 7B,F). Application of exogenous ABA had an effect on recovery from embolism (Figure 7D,F); within 100 min after re-watering, both moderately and severely *not* and *sit* lines showed a significant drop on PLC. This drop was similar to the well-watered plant level, suggesting a full embolism recovery. Application of exogenous ABA on severely stressed plants resulted only in a partial, but significant, drop in PLC level within 100 min, but full recovery was observed within 24 h following re-watering.

**Figure 8:**
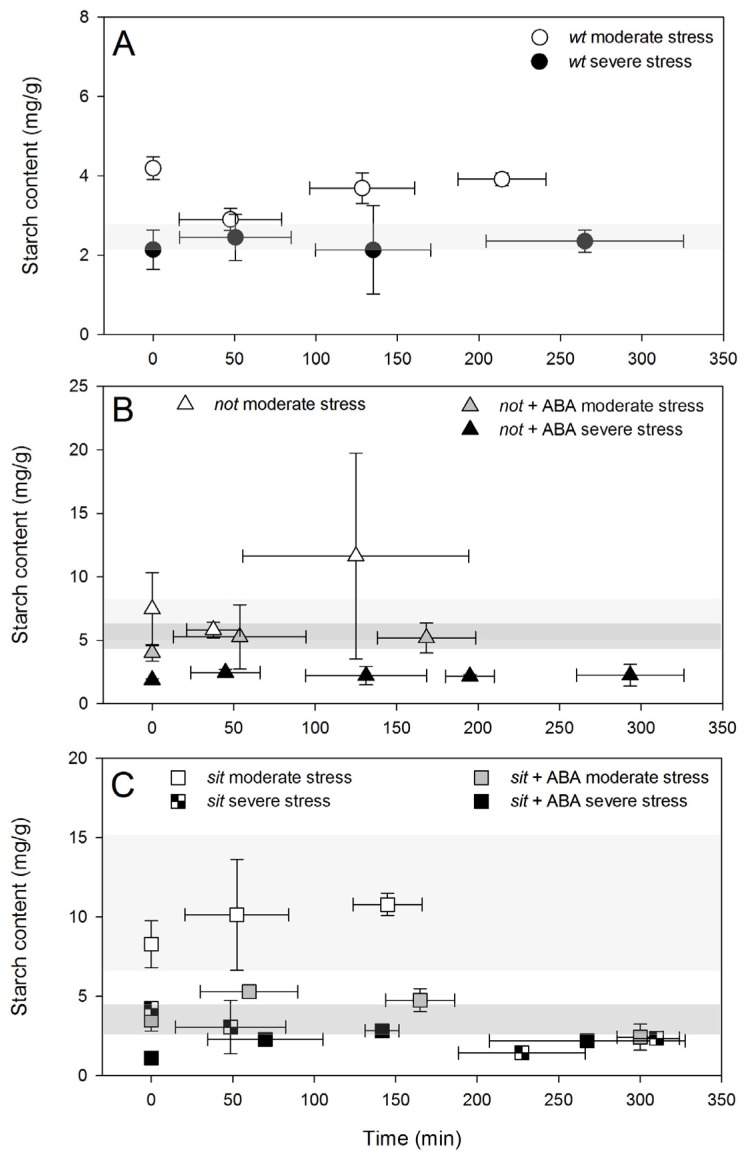
Starch content in petioles following re-watering from moderate and severe stress levels for *wt* (**A**), *not* (**B**) and *sit* (**C**) mutants. Each point is an average of four to six biological samples. Error bars are SD. Shaded grey area width represents average (±SD) starch content, respectively, for well-watered *wt* and ABA-deficient mutants (light grey area) and for well-watered exogenous ABA treated mutants (dark grey area).

The drop in starch content coincides with PLC recovery from embolism. Further recovery marked the return of starch content to initial levels and no significant change in the refilling trend. Severe stress resulted in a significant drop in starch content in all plants and no changes were measured during the recovery time (Figure 8A–C), coinciding with the lack of a significant PLC reduction. Application of exogenous ABA had a significant effect on the starch content of the mutant plants (lower in comparison with untreated mutants), and it was similar to contents measured in wild-type plants. Such reduced content of starch in the petioles of plants treated with exogenous ABA most likely reflects the reductions in stomatal conductance during water stress. However, it might also reflect the difference in carbohydrate metabolism in parenchyma cells that could result in the plant’s ability to use starch as a source of energy for refilling.

## 3. Discussion

Under conditions of water stress, plant survival depends on the ability to maintain a delicate balance between water transport capacity and transpirational loss [11,36]. By allowing for adjustment of transpiration, a variable stomatal conductance (g_s_) coordinates this balance. Stomatal responses to soil drying are assumed to be the result of changes in the concentration of ABA [23], acting in a dual way via biochemical effect on guard cells and a decrease of conductance within vascular tissues [35]. In contrast to wild-type plants, ABA-deficient mutants are typically “wilty”, due to maintenance of a high stomatal conductance, even under well-irrigated conditions [20]. Indeed, both *not* and *sit* mutants were unable to significantly reduce g_s_ until the leaf water potential dropped below −0.9 MPa, which was associated with significant loss of petiole hydraulic conductance, due to embolism formation. Therefore, efficient stomatal regulation is essential to protect xylem from embolism formation [2,37]. In both ABA mutant lines, 50% PLC occurred while stomata were still fully open, and stomata did not shut completely until the occurrence of more than 80% of loss conductivity. Such behavior suggests that ABA-deficient plants were unable to control transpiration rates/leaf water potential in order to avoid significant embolism formation.

Application of exogenous ABA allowed mutant plants to partially increase the sensitivity of stomatal responses to water stress, allowing ABA-treated plants protection from excessive transpiration and delayed the onset of water stress. However, this prolonged application of ABA had no effect on xylem embolism vulnerability, thus suggesting that physical properties of xylem were not affected. It should be noted that application of exogenous hormone resulted in some important changes in the plant allometric proportions. Both mutants produced bigger leaves. This most likely resulted from ABA treatment buffering the water potential of leaves by allowing stomatal control of leaf water potential during the day and closure of stomata at night, permitting the day-night alternations of leaf growth necessary for leaf expansion [38]. The increase of xylem cross sectional area did not match the increase of leaf size area, effectively reducing the Huber value. Thus, it seems that safety of the transport system was secondary to leaf expansion, although biological explanation of this mismatch remains unknown and could be associated with qualitative differences between the effects of root produced ABA *vs.* exogenous ABA [18].

The main motivation for this study was to test whether increased levels of ABA can affect the refilling process in embolized xylem vessels. ABA was shown to influence guard cell osmotic equilibrium, as well as their water status [18–20]. The refilling process relies on changes in osmotic equilibrium of xylem parenchyma cells, and it is possible that ABA is involved in the refilling process during recovery from water stress. Results provide some support for such hypothesis. Moderately stressed *wt* and mutant lines show some level of recovery from embolism within 100 min post re-watering; in *sit* mutants, it was non-significant, and in the *not* mutant, recovery was significant, but did not reach the level of well-watered plants (Figure 7D,F). In both lines, application of exogenous ABA significantly increased the embolism refilling to pre-stress levels. Severely stressed plants were much less responsive to re-watering showing no significant refilling activity, a pattern previously seen in other species [28,39,40]. However, application of ABA significantly induced refilling in both lines within 100 min post re-watering (Figure 7D,F) and full recovery over a prolonged time (Figure 7C,E).

The differences in activity of refilling observed in our experiments could be related to differences in the petioles’ starch content. Content of starch in the petioles of the *wt* plants was lower than in mutants under well-watered conditions. While the level of starch in well-watered mutant plants remains relatively low (5 and 3 mg/g of *not* and *sit* mutants, respectively), in moderately stressed plants the levels increased significantly to 7 and 11 mg/g (*not* and *sit* mutants, respectively). These elevated levels could be related to some limitation of mutant plants in using stored starch to sustain refilling [39,41]. However, in plants exposed to exogenous ABA, starch levels were lower in the range of control plants, suggesting that pools were much more dynamic and potentially used as a source of energy for refilling during recovery. In all severely stressed plants, starch pools were depleted below the control levels, and these low concentrations were associated with limited refilling activity. A parallel further approach involving endogenous ABA stimulation and/or exogenous ABA feedings on mutants with altered starch metabolism could better elucidate the ABA/starch interrelationships during embolism recovery suggested in this work.

## 4. Experimental Section

### 4.1. Plant Material and Growth Condition

Seeds of tomato mutants deficient in ABA, *notabilis* (*not*) and *sitiens* (*sit*), were obtained from the Tomato Genetic Resources Centre, University of California, Davis. The *not* mutants have a defect in a key control step in ABA biosynthesis—the oxidative cleavage of a 9-cis xanthophyll precursor to form the C15 intermediate, xanthoxin [42]. The *sit* mutants are impaired in ABA-aldehyde oxidation and accumulate trans-ABA-alcohol instead of ABA as a result of the biosynthetic block [43]. We used seeds of *Lycopersicon esculentum* with regular ABA synthesis capacity as wild-type plants (*wt*). Seeds were bleached in 2.7% sodium hypochlorite for 30 min, rinsed with deionized water and sown directly into 7 cm diameter, 290 cm^3^ plastic pots filled with potting mix and placed in a glasshouse. Four to six days after germination, individual seedlings were transplanted into 17 cm diameter, 2500 cm^3^ plastic pots and allowed to grow until a minimum of two leaves had expanded fully, upon which the plants were transferred to one gallon pots. Glasshouse temperature was maintained between 20 °C to 30 °C, and natural daylight was supplemented with light from metal halogen lamps if natural light dropped below 500 μmol photons m^−2^ s^−1^. The experimental measurements were carried out on plants that had developed more than 20 fully expanded leaves and often had multiple branching. Flowers were removed from plants to extend the vegetative growth period.

### 4.2. Experimental Design and Water Stress Treatments

A total of 46 tomato plants were used to determine the role ABA plays during the dynamics of embolism formation and repair in stems; of these, 20 plants were wild-type (*wt*) plants, 14 *not* and 12 *sit* mutants. Both mutant lines were divided in two groups. The first group (seven *not* and six *sit* plants) was treated in the same way as *wt*, while the second group was supplied with foliar application of exogenous ABA. ABA was applied biweekly on mutant lines from the seedling stage, and the treatment was stopped seven days before measurements. Treatment with abscisic acid was performed by spraying both sides of the leaves with a solution containing 50 μM of ABA (PhytoTechnology Laboratories, Shawnee, KS, USA).

Of 46 plants, 18 were kept as a control (eight *wt*, three not and two *sit* mutants, three *not* and two *sit* with exogenous-ABA application) and maintained in a well-watered state by watering to field capacity daily at 8 am. The remaining plants were allowed to slowly develop water stress by a gradual reduction in irrigation. By varying the duration of the drought, two levels of water stress were achieved, as confirmed by the measurement of petiole water potential prior to data collection: moderate stress with petiole water potential above −1.0 MPa and severe water stress with petiole water potential below −1.0 MPa, while control plants remained approximately −0.4 MPa. Due to extreme susceptibility to drought, the *not* mutant line was never allowed to reach severe stress levels, due to excessive wilting. All measurements were conducted on control and water-stressed plants concurrently. The physiological measurements (described below) were performed throughout the day, from 9 am to 6 pm, to capture diurnal dynamics of water stress and recovery. Some of the water-stressed plants (12 *wt*, four *not* and four *sit* mutants, four *not* and four *sit* with exogenous-ABA application) were allowed to recover from water stress. These plants were re-watered at 9 am, and measurements started 10 min later and continued with varying intervals until 4 pm. In a few cases, from severely stressed plants, the final measurement was collected the following day at 9 am, allowing for overnight recovery. Samples for ABA and starch content were collected from the same leaves as used in physiological measurements. Control and re-watered plants were used in several consecutive drying periods; however, in such instances we allowed for the development of new leaves before inducing new drought stress.

### 4.3. ABA Extraction and Immunoassay

ABA content was determined in the petioles of control, stressed and recovery plants [44]. Sampled petioles were immediately frozen in liquid nitrogen, lyophilized and ground. The ground dried samples (0.3–0.5 mg) were weighed before extraction in 10 mL cold solvent (80% methanol containing 18 mg L^−1^ butylated-hydroxyl-toluene). Homogenates were kept at 4 °C for 24 h in the dark and centrifuged at 10,000× *g* for 20 min. Supernatants were combined and passed through a Sep Pak C18-cartridge (Millipore Waters, Milford, MA, USA). Methanol was removed under vacuum and the aqueous residue partitioned three times against ethyl acetate at pH 3.0. The combined ethyl acetate supernatant containing the organic fraction was removed under reduced pressure. The residues were taken up in Tris buffer saline (TBS: 25 mM Tris pH 7.5, 150 mM NaCl and 2 mM MgCl_2_) and used for the assay. ABA content was analyzed by an indirect enzyme-linked assay (ELISA) using the Phytodetek assay Kit (Agdia, France), according to the manufacturer instructions. Change in absorbance, derived from the reaction with the substrate, was read at 405 nm using a micro-plateauto-reader (Benchmark micro-plate reader, BioRad, Hercules, CA, USA), and percentage of binding was calculated using established procedures by the protocol included in the kit.

### 4.4. Anatomical Analysis

Xylem area was analyzed on the basipetal part of the petiole below all leaflets. Free-hand cross sections were taken and stained for a few seconds in a diluted Safranin solution, then rinsed in tap water for few minutes and cross sections imaged with a compound microscope. Xylem area was later determined on digital images using ImageJ software (http://rsbweb.nih.gov/ij/). Leaf fresh weight was measured and leaf area determined using a flatbed scanner. Leaves were then dried in an oven for 72 h at 90 °C, after which the dry weight of these tissues was recorded. The Huber value was expressed as xylem area (mm^2^) divided by the leaf area (m^2^).

### 4.5. Measurements of Stomatal Conductance

Stomatal conductance was measured using a porometer (LiCor 1600) on fully expanded leaves. Control and water stressed plants were measured from 9 am to 6 pm immediately before collecting leaflets for petiole water potential; the recovery from stress was measured starting within 10 min of irrigation (9 am) and continued until 4 pm. Petiole water potential was measured on leaflets collected from leaves later used for the percent loss of conductivity (PLC) determination. All leaflets were covered with aluminum foil and placed in a humidified plastic bag for 15 min prior to excision. After excision, leaves were allowed to equilibrate for additional 10 min before the water potential was measured using a Scholander-type pressure chamber (Soil Moisture Equipment Corp., Santa Barbara, CA, USA).

### 4.6. Measurements of Petiole Hydraulic Conductivity

Following the determination of petiole water potential, petiole hydraulic conductivity was measured using an approach described previously [28]. Briefly, sections of petioles (~2 cm long) were cut under water directly from the intact plants. The initial hydraulic conductance (*k*_i_) of each petiole segment was measured gravimetrically by determining the flow rate of filtered 10 mM KCl solution. A water source was located on a balance (Sartorius ± 0.1 mg) and connected to the petiole by a plastic tube. The petiole was submerged in a water bath with a water level ~10 cm below the level of water on the balance. After a steady flow rate was reached (within a few minutes), the tube connecting the petiole to the balance was closed and a bypass tube was used to push water across the segment under ~0.2 MPa of pressure for approximately 20 s to remove embolism. The majority of vessels in tomato petioles are longer than 2 cm [45], thus removal of air from embolized vessels did not require prolonged air dissolution, but simply had to be pushed out [46]. Petiole conductance was then re-measured to find maximum conductance (*k*_max_). The percent loss of conductance (PLC) was calculated as:

(1) $$\text{PLC}=100\times (k_{\text{max}}-k_{\text{i}})/k_{\text{max}}$$

### 4.7. Curve Fitting

Changes in stomatal conductance (g_s_) and PLC in response to petiole water potential were fitted with a four parameter logistic curve. This curve is also known as the response to “dose”: PLC or (g_s_)= min + (max − min)/(1 + (Ψ/EC_50_)^Hillslope^). Plant water potential is treated as the dose, and both PLC and g_s_ behave in a typical dose-response way. EC_50_ is the parameter describing 50% change of the curve between maximum (max) and minimum (min) values. Hillslope describes the slope of the curve at the inclination point. In order to compare different treatments and tomato lines, we used statistically significant differences between EC_50_ parameters.

### 4.8. Starch Analysis

The collected petioles were ground to a fine powder in liquid nitrogen with a mortar and pestle. Starch content was measured as described previously using the STA-20 (Sigma-Aldrich, St. Louis, MO, USA) starch assay kit [41]. Briefly, the soluble sugars were extracted with 80% ethanol for 10 min at 85 °C with constant shaking. Then, the sample was centrifuged at 10,000× *g* for 20 min. The supernatant was discarded, and the resulting pellet was washed three times with 80% (*v*/*v*) ethanol. Then, the pellet was immersed in boiling water for 5 min and digested with α-amylase and α-amyloglucosidase, according to the manufacturer’s protocol. The starch content was determined from the amount of released glucose, which was assayed colorimetrically using a glucose oxidase-mediated method (STA-20, Sigma-Aldrich, St. Louis, MO, USA), and the absorbance was read at 540 nm. Starch concentrations were expressed per fresh biomass.

## 5. Conclusions

The goal of this study was to determine whether ABA influences the ability of plants to refill embolized xylem. We did find some support for this notion, as the presence of exogenous ABA resulted in a significant increase of the refilling capacity of moderately stressed ABA deficient tomato mutants, and stimulated partial recovery in severely stressed plants. Presence of ABA also changed the petioles’ starch pool, suggesting that ABA-deficient mutant plants accumulated starch in petioles to higher levels and possibly had difficulties in reengaging the stored energy to sustain refilling.
